# A European arena for joint innovation in healthcare: The Platform for Innovation of Procurement and Procurement of Innovation (PiPPi)

**DOI:** 10.3389/fpubh.2022.1000590

**Published:** 2023-01-12

**Authors:** Margaret R. Andrews, Preston A. Long, Martina Ahlberg, Fred Balvert, Rossana Alessandrello, Agnese Lazzari, Maarten M. Timmermann, Mariet Nouri Janian, Minerva Rantaniska, Ann Spence, Peter Söderman, Victòria Valls-Comamala, Tanja Stamm

**Affiliations:** ^1^Institute of Outcomes Research, Center for Medical Statistics, Informatics, and Intelligent Systems, Medical University of Vienna, Vienna, Austria; ^2^Center for Innovation, Karolinska University Hospital, Stockholm, Sweden; ^3^Erasmus MC, University Medical Center Rotterdam, Rotterdam, Netherlands; ^4^Innovation Unit, Agency for Health Quality and Assessment of Catalonia (AQuAS), Barcelona, Spain; ^5^Transformation, King's College Hospital NHS Foundation Trust, London, United Kingdom; ^6^Procurement, Erasmus MC, University Medical Center Rotterdam, Rotterdam, Netherlands; ^7^Center for Advanced Technology in Health and Wellbeing, IRCCS Ospedale San Raffaele, Milan, Italy; ^8^Teaching, Research and Development, Helsinki University Hospital, Helsinki, Finland; ^9^Vall d'Hebron University Hospital, Barcelona, Spain; ^10^Health Services Research Group, Institut de Recerca Vall d'Hebron, Barcelona, Spain

**Keywords:** health innovation, digital health, innovation procurement, health policy, Community of Practice, university hospital

## Abstract

By 2000 the European Union (EU) had recognized that its innovation capacity was underperforming in comparison to similar competitors and trading partners. Although the EU has made an effort to stimulate public research and development (R&D) through policy tools like Pre-Commercial Procurement (PCP) and Public Procurement of Innovation (PPI), starting with the 2000 Lisbon strategy and continuing through the 2021 updated Guidance on Innovation Procurement, there has remained a gap in knowledge of and use of these tools, in particular within healthcare. The past decades have seen an explosion in the number and use of digital technologies across the entire spectrum of healthcare. Demand-driven R&D has lagged here, while new digital health R&D has largely been driven by the supply side in a linear fashion, which can have disappointing results. PCP and PPI could have big impacts on the development and uptake of innovative health technology. The Platform for Innovation of Procurement and Procurement of Innovation (PiPPi) project was a Horizon 2020-funded project that ran from December 2018 to May 2022 with a consortium including seven of Europe's premier research hospitals and the Catalan Agency for Health Information. To promote PCP and PPI, PiPPi established a virtual Community of Practice (CoP) that brings together all stakeholder groups to share and innovate around unmet healthcare needs. This perspective presents a brief history of PCP and PPI in Europe with a focus on digital innovation in healthcare before introducing the PiPPi project and its value proposition.

## Introduction

### Two decades of innovation and public procurement in the European Union

By the early 2000's the European Union (EU) had recognized that its innovation capacity was underperforming in comparison to similar competitors and trading partners (1). The 2000 Lisbon strategy for Growth and Jobs (2) aimed to address this gap, with a goal of making Europe “the most competitive and dynamic knowledge-based economy in the world, capable of sustainable economic growth with more and better jobs and greater social cohesion.” The strategy already highlighted the potential for a digital information society to foster improvements from the individual to the international level. The 2005 revised Strategy explicitly included investment in innovation as a focus area (3).

By this time the value of procurement of research and development (R&D) had already been globally demonstrated in multiple fields and further evidence was emerging especially in connection with societal needs and goals (1) (as opposed to the defense and aerospace sectors, which have always dominated R&D procurement). From 2005–2007 several independent expert reports (4) and European Commission communications (1) came out that specifically highlighted public procurement for R&D as a tool to increase the EU's innovation competitiveness, and formally introduced the pre-commercial procurement (PCP) policy tool as a method for public bodies to procure innovation.

In 2010, following the global financial crisis of 2007–2008, the EU released the Europe 2020 (5) strategy for the next 10 years, “A European strategy for smart, sustainable and inclusive growth,” which included the “Innovation Union” as a flagship initiative (6). Member States were directed to engage in public R&D procurement and the EU committed to provide further resources. These resources materialized in the 2011 “Green Paper on Modernisation of EU Public Procurement Policy” (7) and the subsequent 2014 Procurement and Concessions Directives (8). From this time PCP and public procurement of innovation (PPI) were established as tools that could be used by public bodies to buy solutions for identified market gaps. These tools stimulate innovation from the demand side and encourage increased involvement of end-users in the requirements specification and development of solutions, which is a reversal of the more typical supply-side-driven R&D.

However, despite this encouragement and support from the Commission, PCP and PPI continued to be underutilized by Member States. In 2018, the EU issued a new Guidance on Innovation Procurement (9) that built on the 2014 directives. This Guidance was further updated in 2021 (10) in response to the COVID-19 pandemic.

From 2014–2020 the EU directed 80 billion € in funding to identified development goal areas through the Horizon 2020 Programme for Research and Innovation (11). At the time it was the largest such program for international research collaboration, only surpassed by the next iteration, Horizon Europe, starting from 2021. Beyond simply providing funding for innovative R&D, these efforts in fact explicitly provide funding for PCP and PPI actions.

A similar and complementary trend is that of value-based procurement (VBP) (12, 13). The VBP model shifts the goal of procurement from simply low cost per volume to one that considers other outcomes, longer-term results, and wider impacts. This model also emphasizes the need for demand- and supply-side collaboration from the early stages of procurement processes (14). Where PCP and PPI are the European policy tools that public bodies can use for purchasing, VBP is a model that may direct the way the tender is awarded and evaluated.

Of course, it should be considered that diverse opinions on the appropriate balance of public vs. private spending exist (15, 16), and in practice EU member states vary widely in total public expenditure as a percentage of GDP. In 2019, the public spending ratio ranged from over 50% in France, Belgium, and some of the Nordic countries, to ~35%+ in several Eastern European countries, to 32.8% in Switzerland, and 24.2% in Ireland (17). The real utility of the public procurement tools presented here therefore depends partially on location. However, public investment in R&D is clearly also needed to fully optimize innovation capacity, so these tools represent an important opportunity even within public-private mixes weighted toward the private side.

### Innovation procurement in the healthcare context

The healthcare sector is one area where demand-driven R&D has lagged, in particular when considering public hospital activity, and even more so when looking at PCP and PPI actions. However, great potential for fostering healthcare innovation using these policy tools has existed for some time. The EU has used the aforementioned directives and communications, as well as the Horizon funding programs, to steer healthcare actors in this direction. One subfield of healthcare where PCP and PPI could provide great benefits is digital health, both the development of cutting-edge technologies, as well as increasing the adoption of those technologies.

The past decades have seen an explosion in the number and use of digital technologies across the entire spectrum of healthcare. They run the gamut from inexpensive, low-tech solutions to state-of-the-art products that make up significant portions of healthcare costs and budgets. From an organizational perspective, where the latter type of product is more often in question, implementing medical technologies that balance cost and utility is a strategic priority (12, 18).

New digital health R&D has largely been driven by the supply side in a linear fashion (19, 20). An unfortunate side effect of this can be that product uptake, adoption, and diffusion fall short of expectations, which is not uncommon, and is concerning given the large investments in developing and purchasing these products. Collaborative innovation involving all relevant stakeholder groups is still the exception not the norm, so disconnects can exist between the real needs of end-users and the solutions offered by digital technology developers (21, 22). The EU's efforts to increase dialogue between suppliers and purchasers and to stimulate innovation in healthcare through policy tools like PCP and PPI are one macro-level response to this.

Finally, it should also be mentioned that medical procurement and innovation has been greatly influenced by the COVID-19 pandemic. Short-term adjustments included increased procurement efforts for personal protective equipment (23) and medical devices like ventilators (24), as well as EU-level modifications to the formal innovation procurement process in order to streamline and shorten time requirements (25). A greater focus on digital solutions and eHealth models is expected to be a lasting change in the field, however (26–28).

### Current state of affairs

Although it is clear that a concerted public policy effort has gone into stimulating innovation performance in the EU, there remains a gap in the actual knowledge and usage of the PCP and PPI tools. A small number of successful models, such as Campania's regional “One Health” approach (29), and isolated examples can be found, primarily of projects funded through the Horizon 2020 program (30), but innovative procurement still remains the exception. This is especially true when considering a subpopulation of university hospitals. Although these organizations themselves produce some of Europe's leading breakthroughs, they are not known for innovative procurement to address the needs of their own employees and patients. Even when it has been done, these actions are rarely cross-border in nature.

This is problematic, especially in the realm of digital health innovations. Healthcare is increasingly both digital and international in nature. It is no longer sufficient to simply purchase off-the-shelf digital solutions that do not meet real needs, just as solution interoperability and data standardization are crucial for long term success. The status quo, however, has not yet adjusted to these modern realities, leaving healthcare with silo-ed stakeholder groups and technology islands.

To explicitly address this gap, the Coordination and Support Action “Platform for Innovation of Procurement and Procurement of Innovation” received Horizon 2020 funding to build a Community of Practice to bring healthcare stakeholders together to better engage in procurement of innovation. Project outputs, including this perspective, represent the compiled results of over 3 years of qualitative and quantitative data collection from internal and external actors, including expert interviews, workshops, surveys, process mapping, literature review, and KPI analysis, among others.

### Platform for Innovation of Procurement and Procurement of Innovation (PiPPi)

The Platform for Innovation of Procurement and Procurement of Innovation (PiPPi) was an international consortium project funded by the European Union's Horizon 2020 research framework program that ran from December 2018 to May 2022. The project consortium included Karolinska University Hospital (Sweden), Erasmus MC (the Netherlands), King's College London NHS Foundation Trust (UK), Vall d'Hebron University Hospital (Spain), San Raffaele Hospital (Italy), the Catalan Agency for Health Information, Assessment and Quality (Spain), Helsinki University Hospital (Finland), and the Medical University of Vienna (Austria). The overarching goal of the project was to capture unmet needs of university hospitals and to identify opportunities for innovation in digital health and care services.

Based on the combined experiences and knowledge bases of leading research hospitals, the project produced a toolset to assist healthcare actors to identify unmet needs and successfully elaborate them into a formal project plan for a PCP/PPI. This toolset accompanies the PiPPi Community of Practice (CoP), which is a virtual platform (pippi-platform.eu) bringing together all critical stakeholder groups to facilitate the procurement of innovation and the innovation of procurement. The platform was first made available as a beta release in October 2021 and was officially launched in April 2022. In the first year post-project it will continue to be governed by a related consortium.

## Discussion

Platform for Innovation of Procurement and Procurement of Innovation's value proposition rests on several points. First, the platform combines the increasingly popular CoP model with an emphasis on PCP and PPI policy tools to foster healthcare innovation in a way that is unique. A barrier to the use of procurement of innovation at hospitals is low knowledge of and proficiency in using this technique, especially among the key stakeholder groups patients and clinicians. The virtual CoP format allows stakeholder groups to take advantage of modern technologies to network and find commonalities across regions. PiPPi brings stakeholders together to collaborate to understand and define the problems that we all face. The process and related tools that are the results of the project lower the barrier to entry for this type of work.

Second, the CoP provides an opportunity to better aggregate demand and drive the innovation process from the very beginning, which increases the likelihood of successful adoption in the future. As some of Europe's leading research hospitals, the founding members are in a unique position to identify truly unmet needs for which no good market solutions exist and to influence the most beneficial development of solutions.

Lastly and most importantly, the PiPPi project and the CoP have made particular efforts to include patients and clinicians into this process, one which typically excludes these ultimate end-users and beneficiaries. While including a medical expert in technology development is often standard practice, the PiPPi platform provides democratic access possibilities to clinicians of participating institutions. Under the PiPPi model, a clinical champion is a requirement to successfully develop a project plan and move toward PCP/PPI. In addition, the interest of patients and citizens in shaping not only specific interventions, but also the future of healthcare is real and of personal significance, however it is not standard to include these actors until the final stages of testing new solutions. Patient and citizen involvement in unmet need specification is critical to the PiPPi process. During the project a dedicated 12-person Patient and Citizen Advisory Group was established and collaborated on project actions and has advised on sustainable involvement beyond the project end, which will take the form of an overarching patient/citizen working group. The high level of involvement of these stakeholder groups sets PiPPi apart.

## Conclusion

By bringing together all stakeholder groups - with particular value seen from the involvement of patients - in a virtual community devoted to sharing expertise and co-creation, the PiPPi platform evolves the state of European innovation procurement. Although the efforts of the last two decades to advance procurement of innovation in Europe have produced underwhelming usage of PCP and PPI, the stakeholder collaboration made possible by the CoP offers great potential for the future. Further scientific research is needed not only to demonstrate the impact of the PiPPi CoP, but also to elaborate the field of innovation procurement.

## Data availability statement

The original contributions presented in the study are included in the article/supplementary material, further inquiries can be directed to the corresponding author.

## Author contributions

MRA and PAL wrote the first draft of the manuscript. All authors contributed to conception and design of the study, manuscript revision, read, and approved the submitted version.
